# Conservative Sensor Error Modeling Using a Modified Paired Overbound Method and its Application in Satellite-Based Augmentation Systems

**DOI:** 10.3390/s19122826

**Published:** 2019-06-24

**Authors:** Yan Zhang, Zhibin Xiao, Pengpeng Li, Xiaomei Tang, Gang Ou

**Affiliations:** College of Electronic Science, National University of Defense Technology, Changsha 410073, China; 18991679328@163.com (Y.Z.); lipp0808@163.com (P.L.); txm_nnc@126.com (X.T.); ougangcs@gmail.com (G.O.)

**Keywords:** error modeling, conservatism, overbound, satellite-based augmentation system

## Abstract

Conservative sensor error modeling is of great significance in the field of safety-of-life. At present, the overbound method has been widely used in areas such as satellite-based augmentation systems (SBASs) and ground-based augmentation systems (GBASs) that provide integrity service. It can effectively solve the difficulties of non-Gaussian and non-zero mean error modeling and confidence interval estimation of user position error. However, there is still a problem in that the model is too conservative and leads to the lack of availability. In order to further improve the availability of SBASs, an improved paired overbound method is proposed in this paper. Compared with the traditional method, the improved algorithm no longer requires the overbound function to conform to the characteristics of the probability distribution function, so that under the premise of ensuring the integrity of the system, the real error characteristics can be more accurately modeled and measured. The experimental results show that the modified paired overbound method can improve the availability of the system with a probability of about 99%. In view of the fact that conservative error modeling is more sensitive to large deviations, this paper analyzes the robustness of the improved algorithm in the case of abnormal data loss. The maximum deviation under a certain integrity risk is used to illustrate the effectiveness of the improved paired overbound method compared with the original method.

## 1. Introduction

Error modeling is the basis of sensor system design. In many fields that emphasize safety, reliability, and integrity, error modeling tends to pay more attention to the tail characteristics of error. It is necessary to ensure that the error model has a thicker tail than the real error distribution so that our hypothesis testing, anomaly detection, and performance analysis process based on the error model can meet the integrity requirements. Especially in the safety-of-life field (such as aircraft precision approach, etc.), conservative error estimation is more important.

As a “patch” of traditional global navigation satellite systems (GNSSs), the basic purpose of a satellite-based augmentation system (SBAS) [1] includes three aspects. The first is to provide the differential correction parameters of the ephemeris and clock error and the ionosphere delay error to improve the positioning accuracy. The second is to increase the number of available satellites and improve the DOP (dilution of precision) value by adding additional GEO (Geostationary) satellites as range sources. The third is to broadcast the corresponding confidence intervals of the differential corrections, that is, the integrity parameters of user differential range error (UDRE) and grid ionospheric vertical error (GIVE), to provide integrity service for the users (mainly aircraft users) in a wide range. Among them, the most important purpose of SBAS is to improve the integrity application of satellite navigation in the process of aircraft precision approach.

SBAS integrity is based on the fault tree model [2,3]. The fault tree helps system builders sort out all kinds of risks and threat models that may cause integrity anomalies. The corresponding fault elimination method is designed (fault elimination here refers to anomaly detection, variance inflation, and other means to reduce or eliminate the impact of the fault on system integrity). Basically, fault tree includes a fault branch and a fault-free branch. The model parameter estimation and protection level (PL) calculation are all carried out under the fault-free branch. The fault branch is mainly responsible for detecting abnormal conditions through various types of monitors and generating alarm marks in time. In this paper, under the framework of the fault-free branch, an efficient and conservative sensor error modeling method is studied to make sure that it can meet certain system risk performance requirements at the same time to achieve a more accurate simulation of the real distribution characteristics.

Many physical phenomena in nature show the characteristics of a Gaussian distribution due to the additive action of a large number of independent random variables. The error model in the form of a Gaussian distribution is widely used. SBAS integrity-related concepts, including error model parameter estimation and protection level equation, are all based on the assumption of a zero mean Gaussian distribution [4]. Gaussian assumptions have the advantage of simple forms of broadcast parameters, a small amount of data, and convenient user calculation. But in fact, the data are not completely in line with Gaussian distribution characteristics, or do not have enough data to support the verification of the distribution (especially the tail characteristics) of the model [5]. Scholars have carried out research and proposed a series of overbound methods to solve this modeling problem.

The overbound method actually involves two aspects: one is the relationship between the overbound function and the actual distribution, and the other is the specific form to describe the overbound function. Here, we use an overbound function instead of an overbound distribution to emphasize that it is not required to be a probability distribution function (PDF).

**Figure A1:**
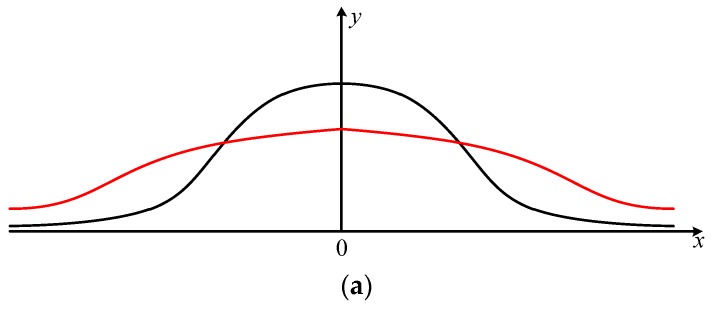

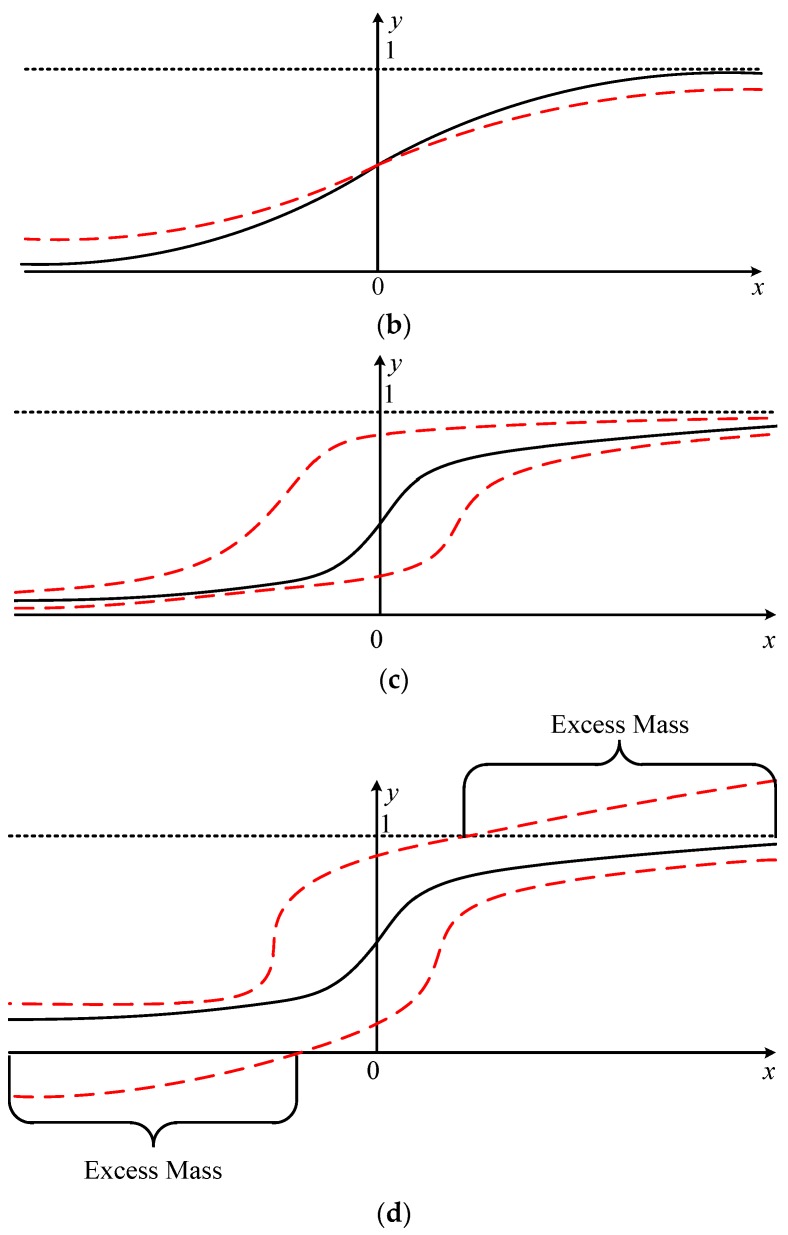
(**a**) Tail overbound. (**b**) CDF overbound. (**c**) Paired overbound. (**d**) EMC paired overbound.

As far as the “relationship” is concerned, it is mainly used to explain what characteristics the established error model should have in order to meet the conservative requirements in the integrity application. SBAS integrity describes the relationship between the actual position error and the protection level calculated by the broadcast parameters. It is defined in the position domain, while the distribution characteristics of the error sources are described in the range domain. The overbound property should be obtained after a convolution operation. The first one is called the tail overbound method [6], which makes the overbound function (the error model we established) have a thicker tail by assigning a greater probability to the values exceeding a certain threshold. But because the tail overbound method only describes the tail characteristics of the error distribution, it cannot ensure that the overbound property is still valid in the position domain. In order to solve this problem, a cumulative distribution function (CDF) overbound method [7] is proposed. It requires that the CDF result of the overbound function in the left part is larger than the CDF of the real error. In contrast, the result in the right half is smaller (Figure A1b). However, the establishment of this method requires that the actual distribution (that is, the real error characteristic) is zero mean, symmetric, and unimodal. In fact, the actual distribution characteristic is difficult to determine. The paired overbound method [8,9] is an improvement of CDF overbound. It uses two overbound functions to model the error distribution characteristics from the left and right sides, respectively, which fundamentally solves the problem of the CDF overbound method being dependent on characteristics of the actual error distribution. At present, it has been applied to the error modeling of practical systems. The above overbound methods all require that the overbound function has the characteristics of a PDF (that is, when the value of a random variable tends to the boundary, the result of the probability distribution tends to one). But in fact, the overbound function does not need to have a real physical meaning—it can be regarded as a conservative way to describe the real error characteristics from a mathematical point of view. Based on this consideration, some scholars have further proposed the EMC (excess mass CDF) method [10]. By removing the restriction that the maximum value of the probability distribution is 1, it effectively reduces the conservatism of the error model and thus improves the availability performance of a SBAS. Although the idea of excess mass was originally used in the paired overbound method, it can also be applied in other methods. The above analyses are aimed at error modeling in the vertical direction and do not take into account the correlation between the errors of epochs; [11] gives research about these two problems with the help of paired Gaussian overbound functions and the autoregressive first-order model.

For the specific form of the overbound function, in addition to the most basic Gaussian distribution, some scholars have proposed Gauss–Laplace [12], NIG (Normal Inverse Gaussian) [13], multi-Gauss [14], and so on. The purpose of their design is to solve the problem of the Gaussian distribution being too conservative in dealing with heavy-tailed characteristics and to improve the availability of the system. However, the non-Gaussian distribution defined in the range domain cannot lead to an analytical error distribution in the position domain, and a series of numerical methods such as feature domain transformation [15] need to be used. This will result in greater computational complexity for the user when calculating the protection level results.

All of the above analyses describe the distribution characteristics of error sources in the range domain (or measurement domain). For a LAAS (local area augmentation system), we can even define the error model in the position domain by making use of the property that the heavy-tailed error will gradually approach the Gaussian distribution after many convolution operations, and the availability performance can thus be improved [16,17]. However, for a wide area differential augmentation system such as WAAS (wide area augmentation system), the geometric difference in satellite observation between users is large and the error correlation is small, so it is difficult to broadcast the error confidence interval directly from the position domain. At present, the error model used in practical systems such as WAAS and LAAS are still Gaussian distributions. With the construction of dual-frequency multi-constellation SBASs (DFMC SBASs), it will provide an opportunity for error modeling in the form of non-zero mean and non-Gaussian distributions [18,19,20,21].

In this paper, a modified paired overbound method is proposed, which still uses the left and right functions to overbound the distribution characteristics of the real error but relaxes the requirements of the overbound function, which no longer has to meet the properties of the probability distribution function. It reduces the calculated protection level while meeting the requirements of integrity and thus improving the availability of the system. In the second part of this paper, the purpose and function of the overbound method are analyzed, and several overbound methods (especially the relationship between the overbound function and the actual error distribution) and their applicable scenarios are introduced in detail. In the third part, a modified paired overbound method is proposed, and its overbound properties of single error source and multi-error source convolution are proved and deduced in detail. In the fourth part, the error data obtained from the real ephemeris are analyzed, and the results show that the modified method plays a positive role in improving availability when compared with the traditional paired overbound method. At the same time, we also show the robustness of the modified method in the case of abnormal data loss. In the last part, the main work of this paper is summarized.

## 2. SBAS Integrity and Error Overbound

### 2.1. SBAS Integrity

SBAS integrity is usually defined by the indicator of integrity risk, which is the probability that the real positioning error of the user exceeds the calculated protection level but does not provide alarm within the specified time (TTA) [22]. In practice, it is difficult for users to obtain their own real positioning errors. According to the assumption of the error distribution of the SBAS, the protection level calculated by using the broadcast integrity parameters can be regarded as the estimation of the real positioning error under the requirement of a certain integrity risk. Whether this estimation is conservative or whether the calculated value can limit the true positioning error under the corresponding integrity risk determines the integrity of the system. The user only completes the calculation of the protection level, and the integrity is not directly related to the result of the protection level. The integrity of a SBAS is determined by the modeling method and the model parameters of all kinds of error sources. To sum up, the properties of SBAS error modeling include: estimation conservatism, convolution invariance, simplicity of calculation, and high efficiency of broadcasting.

#### 2.1.1. Conservatism of Broadcast Error Model

Starting with the definition of the protection level, we take the vertical protection level (VPL) as an example. (The focus on the vertical reflects the tighter demands for vertical navigation, since vertical errors represent a more significant hazard than horizontal errors during landing and since satellite geometry provides poorer position resolution in the vertical direction.) The vertical protection level can be mathematically expressed as [23]:

(1) $$VPL=\left|G_{v}^{-1}\left(P_{HMI}\right)\right|,$$

where *P_HMI_* is the probability of integrity risk, *G_v_* is the CDF of the position error in the vertical direction. *G_v_*^−1^ is the inverse of *G_v_*. SBAS integrity requires that the established error model has a greater tail probability, so that the calculated protection level is larger than the percentile of the real error distribution under an integrity risk. That is,

(2) $$VPL_{m}=\left|G_{m}^{-1}\left(P_{HMI}\right)\right|\geq VPL_{a}=\left|G_{a}^{-1}\left(P_{HMI}\right)\right|,$$

where the subscripts *m* and *a* represent the modeling and actual error distributions, respectively.

#### 2.1.2. Remaining Conservatism after Convolution

As a wide area differential system, the position error distributions faced by users in different SBAS locations are significantly different (mainly because of the difference between observable satellites and the ionosphere delays on the propagation paths). Therefore, the error model should be provided from the range domain (or measurement domain) rather than the position domain. Users calculate the protection level according to the broadcast parameters and their own observation geometry. In the general method of positioning error analysis, the position error and measurement error are in a linear relationship if it is assumed that each satellite’s measurement noise is independent and obeys the same Gaussian distribution. Then the variance characteristic of position error can be estimated by the combination of DOP and satellite UERE (User Equivalent Range Error). (Here, due to length considerations, we do not give a detailed introduction; the relevant content can be found in [24]). For the scene involving the application of integrity, the error distribution cannot be regarded as Gaussian, and the overbound property should be maintained after its transformation from range domain to position domain. Thus, the general DOP-based method cannot be directly used as the estimation of the user positioning error, and it needs to be solved strictly according to the convolution formula.

The vertical error distribution in the position domain can be expressed as

(3) $$G_{v}\left(x\right)=\int_{-\infty }^{x}f_{v}\left(t\right)dt=\int_{-\infty }^{x}f_{e_{1}}\left(t\right)*f_{e_{2}}\left(t\right)*\cdots *f_{e_{n}}\left(t\right)dt,$$

where $f_{e_{i}}\left(t\right)$ represents the error PDF of each pseudo-range source involved in positioning. It is assumed that there is a linear combination of the error sources, and thus the position error PDF is the convolution of all.

SBAS integrity is defined in the position domain, but the parameters that describe the distribution characteristics are broadcast in the range domain. It is necessary to ensure that the conservative model in the range domain still has conservatism after convolution.

#### 2.1.3. Simplicity of User Calculation 

It can be seen from Equations (1) and (3) that the convolution and the inverse of the function need to be completed when users calculate the protection level. For forms of distribution function other than Gaussian, it is usually difficult to get analytical results. The PL results need to be solved by a series of numerical methods such as characteristic domain transformation [25]. The PL results are calculated at the same time as the position solution results, although the frequency will change with the type of receiver, but the overall time interval is about 1 s. Too large a computational complexity is difficult to accept. Therefore, in the current practical SBAS (including WAAS [26], EGNOS (European Geostationary Navigation Overlay Service) [27], GAGAN (GPS-Aided GEO Augmented Navigation) [28], MSAS (Multi-functional Satellite Augmentation System) [29], BD SBAS (BeiDou SBAS) [30], etc.), Gaussian distributions are used, which is also widely recognized by the international community and written into the SBAS MOPS (Minimum Operational Performance Standard) standard. In view of the conservative problem of the Gaussian distribution when it is used to describe heavy-tail errors, scholars have carried out a large amount of research, which include the Laplace distribution, NIG distribution, GPD (Generalized Pareto Distribution ) distribution, and so on. However, because the key problem of computational complexity has not been solved, it has not been applied in practice. In this paper, we still use the error model in Gaussian form but with an addition of a bias term, except for the original standard deviation parameters. The results show that the bias term can significantly improve the availability performance of the system without increasing the processing complexity of the user. At present, the next generation of DFMC SBAS is under construction, and the relevant interface files are being upgraded. It is a good opportunity for us to modify the SBAS error model.

#### 2.1.4. High Efficiency of Broadcasting 

SBAS uses GEO satellite broadcast differential correction parameters and integrity parameters. The longest interval requirements are different for different types of parameters. The update interval for integrity parameters is usually 6 s. In view of the fact that the current downlink rate of GEO is 250 bps and there are many types of parameters to be broadcast, we should (1) use as simple a model as possible, (2) use as few parameters as possible, (3) describe the correction terms and the integrity terms with as small a range (taking into account the quantitative effect) as possible. Because the real distribution characteristics are difficult to effectively describe and broadcast to users, we have to carry out the overbound method.

### 2.2. Previous Overbounding Methods

As mentioned earlier, the overbound method contains two aspects, one is the relationship between the overbound function and the actual error distribution, and the other is the specific form to describe the overbound function. In general, the “relationship” generalizes how to make the overbound function meet the conservatism requirement in the position domain after convolution, which involves the requirements of (1) and (2) in Section 2.1. The specific model form is closely related to (3) and (4). In this section, we will give a detailed introduction to the existing overbound method (mainly the “relationship”), with emphasizes the applicable conditions of the algorithm and compares the advantages and disadvantages between them.

#### 2.2.1. Tail Overbound

The tail overbound method lets the overbound function contain more probability mass in its tails beyond a certain alert limit than the actual distribution [8], so as to ensure that the result of the protection level calculated by the overbound parameter is greater than the percentile corresponding to a certain integrity risk in the real case. For a certain actual error distribution *g_a_*(*x*) (whose corresponding CDF is *G_a_*(*x*)), the tail overbound CDF is defined as

(4) $$\left\{ \begin{array}{l} G_{0}\left(x=-VPL\right)\geq G_{a}\left(x=-VPL\right)\\ G_{0}\left(x=VPL\right)\leq G_{a}\left(x=VPL\right) \end{array}\right..$$

However, this method is only defined at a specific point. Strictly speaking, it can only be guaranteed that the overbound feature is valid at that point. In addition, this method only focuses on the tail part of the error, so it can only overbound the error from the position domain. It cannot meet the convolution requirements of the actual SBAS (or other systems that need to model and classify the errors) from the range domain to the position domain.

#### 2.2.2. PDF Overbound

In order to solve the problem that tail overbound can only be established at a certain point, the PDF overbound method is put forward. It is defined as,

(5) $$g_{o}\left(x\right)\geq g_{a}\left(x\right)\;\forall \left|x\right|>VAL.$$

This is actually a sufficient and unnecessary condition for the definition of tail overbound in (3). When (4) holds, there must be a satisfaction of (3), and it is true for any |x|>VAL. This means that when the real position error exceeds the vertical alarm limit (VAL), it must be able to ensure that the VPL result obtained by using the PDF overbound method is larger than the real VPL value. The user can determine that the service is “unavailable” after comparing the calculated (or overbound) *VPL_o_* with the VAL, and there will be no hazardous misleading information (HMI) event. However, similar to the tail overbound method, the PDF overbound method also has a serious defect: only the tail part of the error distribution is overbounded, and there are no constraints on the core part. When the error distribution of the sum of multiple random variables is involved, we need to make convolution operations. At this time, there is no guarantee that the overbound property is still valid. In a SBAS, because the description of error characteristics is in the range domain, users need to complete the conversion from the range domain to the position domain, so the above two methods cannot be applied directly.

#### 2.2.3. CDF Overbound

As can be seen from the above analysis, in order to ensure integrity, a greater probability must be assigned to the tail of the overbound function. At the same time, in order to ensure that the conservatism is still valid after the convolution operation, it is necessary to make a definition in the whole value range of variable *x*. The CDF overbound method takes into account both of the above requirements, which are defined as

(6) $$\begin{array}{l} G_{0}\left(x\right)\geq G_{a}\left(x\right),\forall G_{a}<\frac{1}{2}\\ G_{0}\left(x\right)<G_{a}\left(x\right),\forall G_{a}\geq \frac{1}{2} \end{array}$$

However, the establishment of the CDF overbound method needs to make certain assumptions about the real error characteristics and the overbound function, which are required to have the characteristics of zero mean, symmetry, and unimodality. Whether these assumptions are satisfied or not is difficult to verify in practice. This has caused great difficulties in its application.

#### 2.2.4. Paired Overbound

The paired overbound method uses the left and right functions to realize the overbound purpose for a single random variable and the sum of random variables. It is defined as

(7) $$\begin{array}{l} G_{L}\left(x\right)\geq G_{a}\left(x\right),\forall x,\\ G_{R}\left(x\right)\leq G_{a}\left(x\right),\forall x. \end{array}$$

The final overbound function is a combination of the left and right parts. This is the first applicable conservative method to overbound the error of navigation source, which has been widely used in WAAS and LAAS. Different from the previous methods, the paired overbound method first convolutes the overbound functions on the left and right sides separately and then combines them to obtain the final overbound results of the actual error distribution.

All of the above methods require the overbound function to have the characteristic of a probability distribution function, that is, when the independent variable tends to the boundary, the corresponding value of the overbound function tends to 0 (lower boundary) or 1 (upper boundary). But in fact, the overbound function is only a conservative description in mathematics, which itself does not have to meet the physical meaning of CDF. By relaxing the restrictions on the form of the overbound function, the calculation results of the protection level can be further reduced and the availability of the system can be improved. EMC (Excess Mass PDF, EMP) is actually the use of this idea.

The enlightenment can be obtained from the paired overbound method, which is a “convolution before combination” solution, and the application of the excess mass method: our goal is to overbound the error distribution in the position domain. The overbound function in the range domain is only an intermediate result and does not require it to conform to the characteristics of the probability distribution function. Therefore, it can be more flexible to model the error of a single sensor in the range domain (or measurement domain). Based on this idea, this paper improves the widely used paired overbound method from the “relationship” between the overbound function and the actual error distribution.

## 3. Modified Paired Overbound Method and Its Integrity

### 3.1. Modified Paired Overbound Method

The paired overbound method can be applied under arbitrary error distributions, and the problems existing in the hypothesis and verification of the actual error distribution can be effectively avoided. At the same time, it defines the relationship within the whole range of independent variables and can satisfy the convolution requirement when converting from range domain to position domain. However, the left and right parts of paired overbound are required to be greater or smaller than the actual error distribution at the same time, which will make the envelope be too conservative. In addition, the envelope will gradually increase with the increase in convolutions (the number of error sources). As a contrast, the CDF overbound method only takes into account the unilateral characteristics, and the limit function is more approximate to the real error distribution. However, the main problem of CDF overbound is that the actual error distribution must be zero mean, symmetrical, and unimodal. The modified paired overbound method proposed in this paper combines the advantages of CDF overbound and paired overbound. The main idea is to relax the limitation that the overbound function must meet the characteristic of probability distribution and to describe the unilateral relationship. Thus the envelope of the actual error distributions from the left and right overbound functions is better than that of the traditional paired overbound method. In fact, the modified method is the result of “convolution before combination” of the error distributions, which can effectively reduce the overall protection level results and improve the availability of the system.

The following formula gives the description of the relationship between the overbound function and the actual error distribution by the modified paired overbound method.

(8) $$\begin{array}{l} \left\{ \begin{array}{l} G_{L}\left(x\right)\geq G_{a}\left(x\right),\forall G_{a}\left(x\right)<\frac{1}{2}\\ G_{L}\left(x\right)=G_{a}\left(x\right),\forall G_{a}\left(x\right)\geq \frac{1}{2} \end{array}\right.\\ \left\{ \begin{array}{l} G_{R}\left(x\right)\leq G_{a}\left(x\right),\forall G_{a}\left(x\right)\geq \frac{1}{2}\\ G_{R}\left(x\right)=G_{a}\left(x\right),\forall G_{a}\left(x\right)<\frac{1}{2} \end{array}\right. \end{array}$$

**Figure 1:**
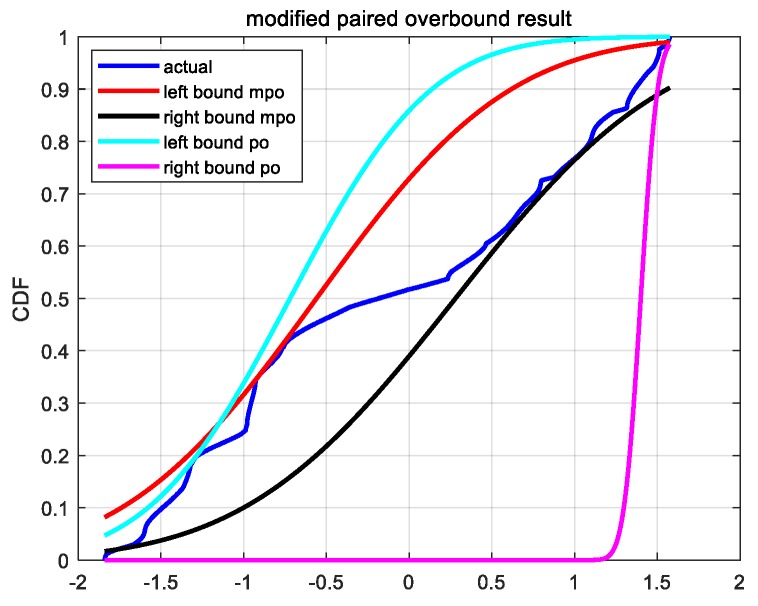
The empirical CDF and the overbound function obtained from the two paired overbound methods. The “MPO” in the legend stands for the modified paired overbound method, the “PO” in the legend stands for the paired overbound method.

In Figure 1, we graphically show the differences between the two overbound methods. We make use of the difference between the precise satellite position provided by IGS (International GNSS Service) and the broadcast satellite position modified by SBAS and obtain the ephemeris error data. Figure 1 shows the ephemeris errors in the *x* direction of the PRN1 GPS satellite. The time is from 2017day121 UT 00:00:00 to UT 12:00:00. The sampling interval is 30 s. More information about the data source and the data pre-processing method is described in Section 4.1.

In Figure 1, the dark blue curve represents the empirical CDF obtained from the actual error samples. The light-blue and light-red curves represent the CDF of the left and right overbound function obtained by the original paired overbound (PO) method, respectively. The red and black curves represent the CDF of the left and right overbound function obtained by the proposed modified paired overbound (MPO) method, respectively. As we can see, the overbound function obtained from the modified method is a better approximation to the empirical error distribution. In addition, we need to give two more explanations about Figure 1. One is that near the 25% quantile (lower left corner in Figure 1), the MPO and PO methods seem to have their own advantages and disadvantages. The other is that the right overbound function of the PO method seems to be particularly poor. For the first one, the reason is that under the definition of MPO (Equation (8)) and PO (Equation (7)), we could have infinite candidates of (*μ*,*σ*). As for which candidate (*μ*,*σ*) is selected, this is strongly related to the optimal criterion. Here, we choose the candidate that has the smallest difference with the empirical CDF. Therefore, at some quartile points, it does not exclude that the overbound CDF obtained from the PO method is closer to the empirical CDF. For the second one, it is actually determined by the characteristics of the empirical CDF, and the thicker the tail of the empirical CDF, the more obvious this phenomenon is. This also verifies the necessity of the MPO method proposed in this paper from another aspect.

The correctness (conservatism for a single random variable and sum of variables) of the modified paired overbound method is explained below.

### 3.2. Overbound Property for a Single Error Source

In this subsection, we will prove that the overbound function obtained by Equation (8) is a conservative estimate of the actual error distribution. That is, it should be explained that the following formula is valid.

(9) $$VPL_{o}=\max\left(VPL_{l}^{o},VPL_{r}^{o}\right)\geq \max\left(VPL_{l}^{a},VPL_{r}^{a}\right)=VPL_{a}$$

The subscripts *l* and *r* represent the left and right overbound functions, respectively, and the superscripts *o* and *a* represent the overbound distribution and the actual error distribution, respectively (here we use *o* instead of *m* in Equation (1) to emphasize that the error estimate is a kind of overbound estimation).

For the left part,

(10) $$P_{HMI}=G_{o}\left(-VPL_{o}^{l}\right)=G_{a}\left(-VPL_{a}^{l}\right).$$

According to the definition in (8), we have that

(11) $$G_{o}\left(-VPL_{o}^{l}\right)>G_{a}\left(-VPL_{o}^{l}\right).$$

Thus, we may have,

(12) $$G_{a}\left(-VPL_{a}^{l}\right)>G_{a}\left(-VPL_{o}^{l}\right).$$

And because the CDF *G_a_* is increasing, we have that

(13) $$VPL_{a}^{l}<VPL_{o}^{l}.$$

Similarly, for the right part we have,

(14) $$VPL_{a}^{r}<VPL_{o}^{r}.$$

Thus, the Equation (9) can be proved.

### 3.3. Overbound Property after Convolution

Here, we prove that the overbound property still holds after the convolution operation. The linear operation includes addition and multiplication operations, and the random variable multiplied by a constant will not affect its distribution. Therefore, here we mainly explain the relationship between the original error distribution and the distribution after the addition operation. Take the case of an addition of two random variables as an example.

(15) $$G_{m+n}\left(x\right)=\int_{-\infty }^{x}\int_{-\infty }^{+\infty }g_{m}\left(t-z\right)g_{n}\left(z\right)dzdt=\int_{-\infty }^{+\infty }g_{n}\left(z\right)G_{m}\left(x-z\right)dz,$$

(16) $$G_{Lm+n}\left(x\right)=\int_{-\infty }^{x}\int_{-\infty }^{+\infty }g_{Lm}\left(t-z\right)g_{n}\left(z\right)dzdt=\int_{-\infty }^{+\infty }g_{n}\left(z\right)G_{Lm}\left(x-z\right)dz,$$

where *m* and *n* represent two random errors, and *L_m_* is the left overbound function. By subtraction, we have that

(17) $$G_{Lm+n}\left(x\right)-G_{m+n}\left(x\right)=\int_{-\infty }^{+\infty }g_{n}\left(z\right)\left[G_{Lm}\left(x-z\right)-G_{m}\left(x\right)\right]dz.$$

From Equation (8), we know that the result of the above equation is always greater than 0. That is to say, when the error distribution is overbounded at the beginning, it is not necessary to overbound the whole range of independent variables as in the traditional paired overbound method, but the final result can still achieve the effect of the traditional one. And the same is true for the right part.

### 3.4. Trade-off between Mean and Standard Deviation

For the paired overbound method, there are two variables: bias and standard deviation. For a certain VPL result, the corresponding error distribution is no longer unique. The conservatism requirement can be satisfied (overbound the existing sampling points) both by increasing the bias term or the standard deviation term. The following figure shows an example.

**Figure 2:**
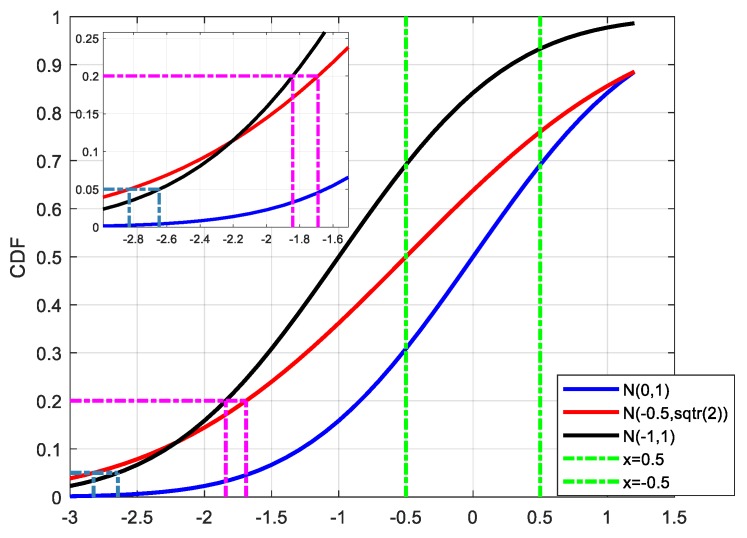
The trade-off between the mean and standard derivation of the Gaussian distribution when the error samples are overbound. The choice of a better overbounding function with higher availability may be different if the integrity risks are settled differently.

In Figure 2, the blue curve represents the actual error distribution, which obeys the standard Gaussian distribution with a mean value of 0 and a standard deviation of 1 (N(0,1), N stands for the Gaussian distribution). Assuming that the samples are randomly sampled from N(0,1) with a range of [−0.5 0.5], the maximum value of the samples is no bigger than 0.5 and the minimum value of the samples is no smaller than −0.5. When doing overbounding, we should make sure that all the samples are satisfied with the definition of the overbound method.

The red and black curves represent the CDF results of N(−0.5,$\;\sqrt{2}$) and N(−1,1), respectively, which both can be regarded as overbound results for N(0,1) with the samples in the range of [−0.5,0.5]. (In this case, the left part is taken as an example.) It can be seen that under the case of P = 0.05, the corresponding percentiles of N(−0.5,$\;\sqrt{2}$) and N(−1,1) are −2.8262 and −2.6449, respectively, and thus the overbound function N(−1,1) will be regarded as a better choice for its smaller percentile result (in its absolute form). However, under the case of P = 0.2, the percentiles of N(−0.5,$\;\sqrt{2}$) and N(−1,1) are −1.6902 and −1.8416, and thus the overbound function N(−0.5,$\;\sqrt{2}$) will have better availability. It can be seen that we may get a different conclusion on the choice of an overbound function under a different probability, and it is inappropriate to evaluate the modeling performance just relying on a few points.

The trade-off relationship between mean and standard deviation in the paired overbound method is found in the literature, but there is no detailed explanation of how we should evaluate the performance of the overbound function at this time. Here, we need to make a detailed analysis of this problem.

The trade-off between *σ* and *μ* is an intuitive external representation, and its inner embodiment is the trade-off between availability and integrity. As shown in Figure 2, for the case where the actual error is heavy-tailed, either increasing the mean or the standard deviation of the Gaussian distribution could achieve the purpose of overbounding, but their performances are different. In the extreme case, the characteristic of the overbound function obtained by increasing the mean value tends to reach that of a step response (the CDF of a pulse function). Suppose that the jump in the CDF of a step response is *x* = *x*_0_. This means that the data with an absolute value bigger than $\left|x_{0}\right|$ are all regarded as “unavailable”. It is similar to the concept of hard cut-off. If *x*_0_ is too small, the availability will be harmed, and if it is too large, it will affect the integrity of the system. Besides, multiple convolutions are involved in the conversion from range domain to position domain. With the increase in convolutions, the problem caused by excessive dependence on the mean of the Gaussian distribution will become more serious. The following figure shows the relationship between the overbound function and the actual error after 1, 2, and 5 convolutions.

**Figure 3:**
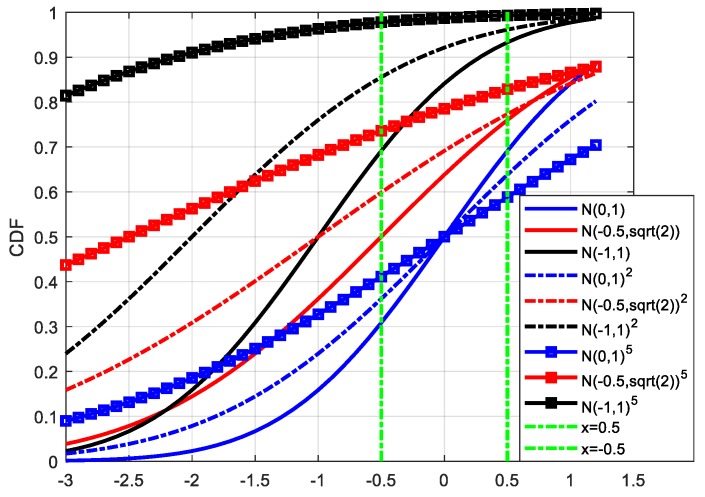
The relationship between the overbound function and the actual error after 1, 2, and 5 convolutions. With the increase in convolutions, the differences between the overbound functions and the actual error distribution increase, and the results tends to reach the CDF of a step response.

In Figure 3, blue, red, and black curves represent the distribution of N(0,1), N(−0.5,$\;\sqrt{2}$), and N(−1,1), respectively. Solid lines, dashed lines, and lines with square markers represent the results after 1, 2, and 5 convolutions, respectively. It can be seen from the figure that with the gradual increase in convolutions, the difference between the overbound function and the actual error is greater, and the distributions are approaching the CDF of a step response, especially for the overbound function with a larger mean value. In this case, although the corresponding percentiles are smaller under some probabilities, it should not be considered a good model of the actual error.

Here, we introduce the concept of *EVPL* to illustrate the overall performance of the overbound function in the whole probability value space, namely [0,1]. It is defined as

(18) $$EVPL=E\left[p_{i}\cdot VPL\left(p_{i}\right)\right],$$

where *p_i_* denotes probability, and *VPL*(*p_i_*) denotes the percentile corresponding to *p_i_* under the standard normal distribution. In discrete form, it can be expressed as

(19) $$EVPL=\frac{\sum_{i=1}^{N}p_{i}\left(K\left(p_{i}\right)\sigma +\mu \right)}{N}=\frac{\sum_{i=1}^{N}p_{i}K\left(p_{i}\right)}{N}\sigma +\mu .$$

Below, we will start with this indicator to analyze the performance differences between the improved method proposed in this paper and the traditional paired overbound method.

## 4. Performance Analysis

### 4.1. Data Collection

In this paper, we take the overbound of satellite position error as an example. The difference between the precise ephemeris data provided by IGS [31] and the broadcast ephemeris which is corrected by SBAS (in this case, EGNOS). The EGNOS augmentation information comes from the EGNOS Message Service (EMS) [32], which is provided by ESA (European Space Agency). The constellation is a GPS constellation. For availability performance analysis, it is assumed that users are located in the ECAC (European Civil Aviation Conference) [33] area, whose longitude range is −40°~40° and latitude range is 20°~70°. Users are distributed in a grid mesh, and the grid resolution is 1° × 1°. The date is from 27 April 2017 (day117) to 3 May 2017 (day123), a total of 7 days. To avoid correlation between the data, the sample interval is selected as 600 s.

### 4.2. Generation of Overbound Parameters

After getting the error samples, we then get the parameters of the overbound function. The detailed procedure is shown in Appendix A. It is mainly divided into the following three parts: overbounding the error in a single direction, finding the worst user location in the range domain. and hypothesis testing of the results.

1. Calculate overbound results in the *x*, *y*, and *z* direction.

We take the *x* direction as an example. For the error sample set of each satellite, the search range and search step of the parameters μ and σ are first set, and we look for a parameter pair (μ, σ) that brings the CDF result closest to the empirical CDF. At the same time, this (μ, σ) must meet the requirement that the overbound function is not less than the empirical CDF. If such a (μ, σ) does not exist, we should then increase the search range until the appropriate parameters are successfully found or the number of searches reaches the setting threshold.

2. Find the worst user location in the service area and obtain the scalar overbound result in the range domain.

When the overbound results are obtained in each of the three directions, if the value in any direction of a satellite is empty, the UDRE of the satellite at that time is marked as “Do not use”; otherwise, the worst user location (WUL) needs to be calculated and the scalar overbound result in the range domain needs to be generated. The methods of calculating the WUL can roughly be divided into two parts: the exhaustive search method and the geometric calculation method [34]. Here, our main purpose is to verify the performance of the modified paired overbound method, so we choose the exhaustive search method for its simplicity.

Specifically, first we need to calculate the observation vectors from the users to satellites. Then we get the corresponding scalar error in the range domain by combining the observation vector with the ephemeris error vector. At last, the WUL vector ${\vec{a}}_{WUL}$, corresponding to the maximum error in the range domain that satisfies the elevation threshold, is found. The overbound parameters (μ, σ) in the range domain can be calculated according to the following equations.

(20) $$\sigma =a_{WUL}^{T}\left[\begin{array}{ccc} \sigma_{x}^{2} & 0 & 0\\ 0 & \sigma_{y}^{2} & 0\\ 0 & 0 & \sigma_{z}^{2} \end{array}\right]a_{WUL}$$

(21) $$\mu =a_{WUL}^{T}\left[\begin{array}{c} \mu_{x}\\ \mu_{y}\\ \mu_{z} \end{array}\right]$$

3. Hypothesis test of the obtained results

The overbound results in the range domain for each satellite at each time can be obtained by the above two steps, which need to be further tested to meet the requirements of false alarm and misdetection. The hypothesis test is based on the fact that an event with a small probability hardly occurs, and if it does occur under a hypothesis, it is reasonable for us to believe that the hypothesis itself is wrong. In the hypothesis test, the conclusion of “reject H0” is more persuasive. “Accept H0” does not mean that H0 must be correct, but that there is no contradiction between the observation and H0. Therefore, if we want to use the hypothesis test method to prove the validity of a conclusion, it is best to raise the H0 hypothesis to “the conclusion is not true”. If it is refused, then the conclusion is credible.

Based on the above considerations, we make that

(22) $$\begin{array}{l} H0:p_{tail}>p_{0},\\ H1:p_{tail}\leq p_{0}. \end{array}$$

where *p_tail_* represents a probability of exceeding a certain *X*_0_, that is, the tail probability for the actual error distribution. *p*_0_ is calculated from the overbound parameters estimated above.

The test statistic *T* is defined as the number of times in the sample set the absolute value of error exceeds *X*_0_. If H1 is true, namely, the actual tail probability *p_tail_* is less than *p*_0_, the number of large deviations should be small. Thus, the rejection domain has the form

(23) T≤C.

We calculate the value of threshold C according to the requirement of false alarm.

(24) $$P_{H_{0}}\left\{reject\;H_{0}\right\}=P\left(T\leq C\right)=\sum_{j=0}^{C}C_{N}^{j}p_{0}^{j}{\left(1-p_{0}\right)}^{N-j}=\alpha$$

Here, we choose *X*_0_ = max(*X*). If the hypothesis test fails, the first step is to return to estimating the overbound parameters, and the search range of μ and σ should be adjusted accordingly.

### 4.3. Availability Performance

#### 4.3.1. Overbound Property for a Single Error Source

First of all, we compare the difference between the overbound functions obtained under the traditional paired overbound method and the modified paired overbound method, and the empirical error CDF. First, we need to define a proper metric to describe this kind of difference. It should at least satisfy the following four characters:

■ Since both the MPO and PO methods are defined from the perspective of the CDF, it is natural for us to define a metric from the point of view of the CDF to describe the difference between the overbound function and the empirical CDF.
■ In order to maintain the overbound property when transferring from range domain to position domain, one of the most basic requirements is that the overbound function should be defined within the entire range of independent variables. Thus, we want to define it not only in some special positions (like the traditional total variation distance [35]), but also **in** the entire range of independent variables.
■ The metric needs to have unique zero points. That is to say, if and only if the overbound function and the empirical CDF are the same, the result of the metric is zero. Otherwise, it will be difficult to define what is the best approximation of the empirical CDF. Thus, some metrics that are based on the PDF (like the Kullback–Leibler divergence [36]) cannot be selected.
■ Because integrity actually places more emphasis on overbounding for the sampling points that are far from the core part and with a very small probability of occurrence, we do not want to weigh the divergence according to its probability. That is the reason why we did not choose a more “standard” Carmer–von Mises [37] distance.

Based on the reasons above, we use the concept of sum of difference (SUMD) to describe the difference, which is defined as

(25) $$SUMD=\int_{-\infty }^{+\infty }\left|G_{o}\left(x\right)-G_{a}\left(x\right)\right|dx=\sum_{i=1}^{N_{bin}}\left|G_{o}\left(x_{i}\right)-G_{a}\left(x_{i}\right)\right|.$$

**Figure 4:**
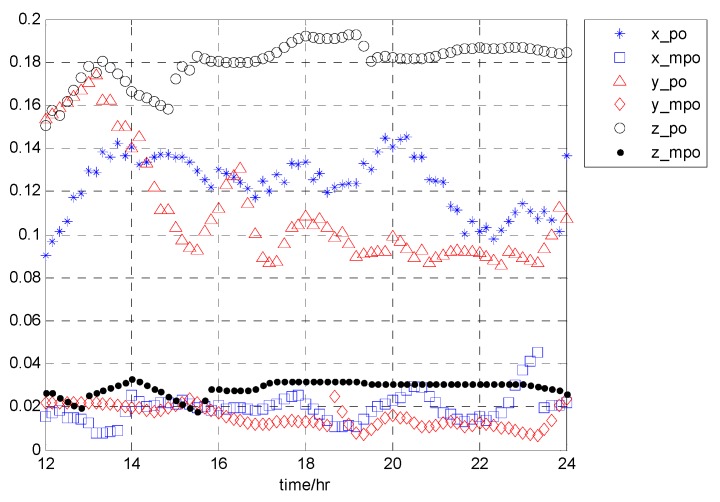
The variation in CDF difference with time (2017day121 UT12:00:00~24:00:00) for the two paired overbound methods, taking the result from PRN1 as an example.

In the following Figure 4, satellite PRN1 is taken as an example to show the variation of the CDF difference with time (2017day121 UT12:00:00~24:00:00) for the two overbound methods. Blue, red, and black curves represent the results in the x, y, and z directions (in the ECEF (Earth Centered Earth Fixed) coordinates frame), respectively.

**Figure 5:**
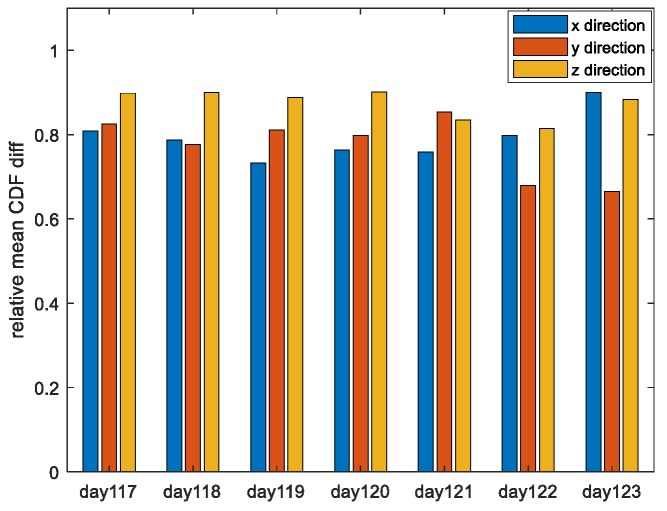
The rate of performance improvement with the MPO method compared to the PO method for all the three directions of the two paired overbound methods. The date is from 2017day117 to 2017day123. The results from PRN1 are taken as an example.

The horizontal axis represents time with a resolution of 1/6 h. The vertical axis represents the SUMD results, which have been averaged over the whole range of independent variables. Using the data of the first 12 h, we estimate the overbound result of the current epoch. The overbound results vary slowly with time. It can be seen that compared with the traditional PO method, the MPO method proposed in this paper can better approximate the empirical CDF in three directions. Furthermore, in Figure 5, we statistic the performance improvement of our proposed PO method under the SUMD index.

The date is from 2017day117 to 2017day123. Taking satellite PRN1 as an example, the rate of performance improvement with the MPO method compared to the PO method for all three directions of the two paired overbound methods is shown.

It can be seen that when the single error source is overbound, the method proposed in this paper can obtain a more accurate approximation of the actual error distribution than the traditional method when the overbound requirements defined in Equations (7) and (8) are satisfied. Specifically, under the experimental conditions given in this paper, the mean difference between the overbound function obtained by the traditional paired overbound method and the empirical error distribution in the x, y, and z directions are 21.09%, 12.29%, and 14.06%, respectively. (The results of other valid satellites are similar to those of PRN1.) The mean differences obtained by the modified paired overbound method are 4.48%, 2.73%, and 1.79% for the three directions. The increase is not less than 70%.

#### 4.3.2. Overbound Property in the Position Domain

**Figure 6:**
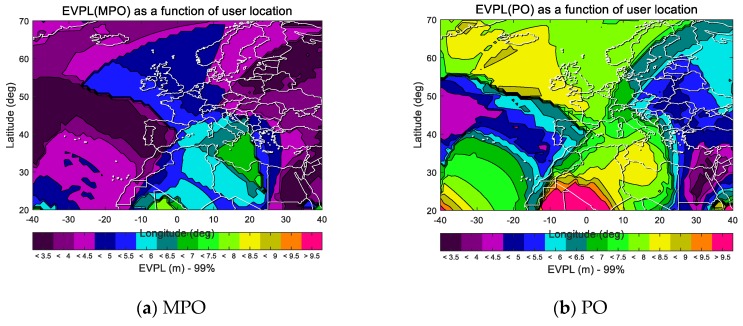
The *EVPL* (99%) results of users in the ECAC (European Civil Aviation Conference) area in the case of 2017day121: (**a**) the *EVPL* (99%) results of MPO method; (**b**) the *EVPL* (99%) results of PO method

Above, the performances of the two paired overbound methods on a single error source are analyzed in the range domain. Using the MAAST tool [38], we further compare the difference in availability between the two overbound methods (that is, the error distributions of multiple error sources after convolution). Under the experimental settings given in Section 4.1, the following Figure 6 show the EVPL results of users in the ECAC area at a 99% time ratio in the case of 2017day121. The definition of the EVPL metric and the reasons for using it for availability evaluation are described in detail in Section 3.4.

The indicator relative EVPL (REVPL), measuring the ratio of performance improvement, is defined as

(26) $$REVPL\left(x_{i};pct\right)=\frac{EVPL_{po}\left(x_{i};pct\right)-EVPL_{mpo}\left(x_{i};pct\right)}{EVPL_{po}\left(x_{i};pct\right)},$$

where *x_i_* represents the user location, *EVPL* is the indicator defined in Section 3.4, and the subscripts *po* and *mpo* correspond to the traditional paired overbound method and the modified paired overbound method. *pct* represents the percentage of time and is used to obtain the *EVPL*(*pct*), which is the percentile under a probability of *pct*. The REVPL result is shown below.

Statistics are provided to show the proportion of users with different rates of growth.

**Figure 7:**
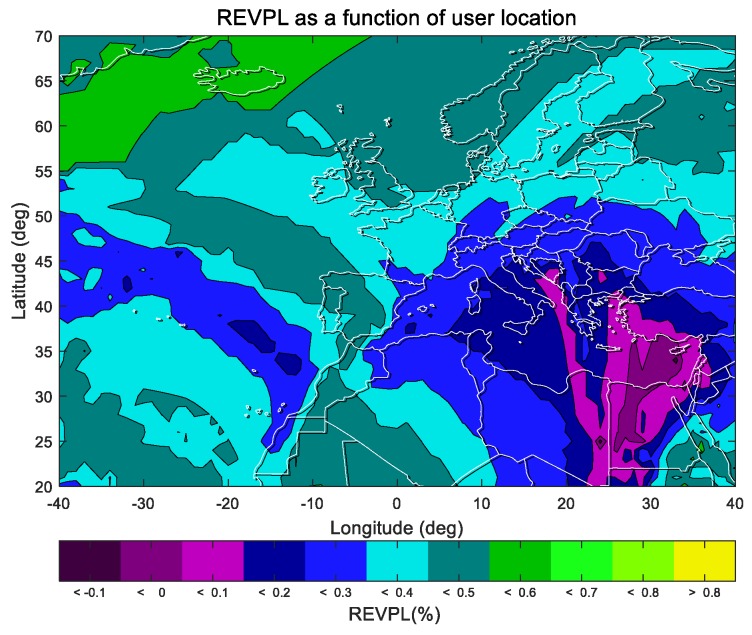
The relative EVPL (REVPL, 99%) results for the date of 2017day121. It shows that in about 99% of the region, using the modified paired overbound method can achieve better EVPL results than the traditional one.

Table 1 describes the difference between EVPL results obtained from the MPO and PO methods (REVPL in Equation (26)) for all the users located in the ECAC area. The first row in the table represents the intervals in which the REVPL results are located. The second row represents the percentage of users falling into the corresponding interval in the ECAC area. According to the definition of Equation (26), if the REVPL value is greater than 0, the smaller the EVPL results from the MPO method, which means a higher availability. It can be seen that in about 99% of the regions, the results of the REVPL are greater than 0, which indicates the better availability performance of our proposed MPO method. In Table 2, Further statistics are provided on the proportion of users in intervals with different rates of growth during 2017day117 ~ 2017day123. Here, the interval given in Figure 7 is adjusted to merge some of the intervals with smaller proportions.

**Figure 8:**
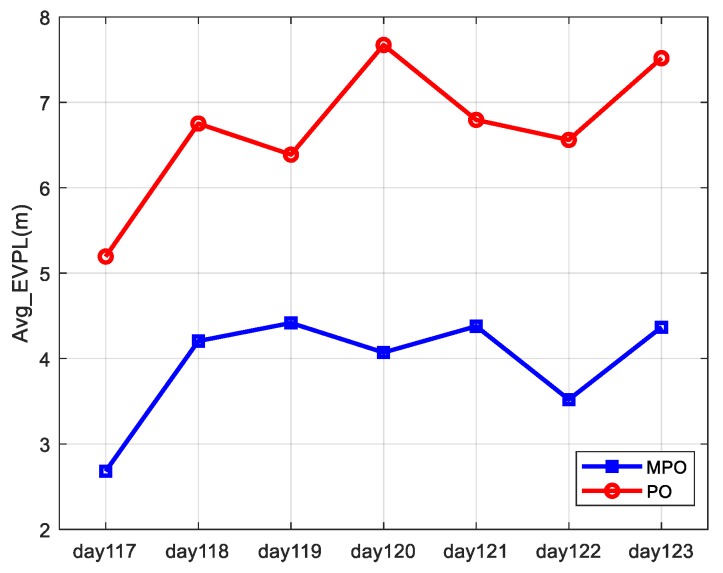
The daily average EVPL (99%) results for all users in the service area.

The average EVPL (99%) results for all users in the service area are analyzed as an overall description of the availability of the two paired overbound methods. As shown in Figure 8, the horizontal coordinates are the analysis date, and the vertical coordinates are the average EVPL (99%) results. The blue and red curves represent the results obtained by the MPO and PO methods.

The daily average EVPL (99%) results are not exactly the same because the distribution of satellite error corrected by SBAS is different on different dates. Comparing the results obtained by the MPO and PO methods, we can see that the two results have similar trends, indicating that both of them can correctly reflect the actual error characteristics but that the MPO method can lead to a better availability performance.

### 4.4. Robustness for Large Errors

This section will show that in addition to availability, the improved paired overbound method proposed in this paper is more robust to large deviations. That is, assuming that for a certain set of error samples, pairs of overbound parameters are obtained by using the MPO and PO methods described above, the parameter (*σ*,*μ*) corresponding to the MPO method can “tolerate” larger deviations. The content of this section can be divided into three parts. First, we define the robustness of overbound to large deviations (here, a large deviation means that the value is far from core of the distribution). Secondly, the robustness of the overbound results is quantitatively described by the index of “maximum allowable deviation”. Finally, the performance difference between the proposed MPO method and the traditional PO method in terms of robustness is compared by using the defined “maximum allowable deviation”. 

Generally speaking, robustness refers to the nature of a system that can still work properly under abnormal circumstances. For conservative error overbounding, because SBAS integrity places more emphasis on the conservative modeling of sample data that are far from the core and have a small probability of occurrence, the influence of the loss of a “large deviation” sample on the result is far from the influence of the loss of a sample that is near the core of the distribution. Thus, we define the robustness of conservative error overbound as: when a “large deviation” sample is lost, the overbound property could still be guaranteed by using the overbound result that is generated from the remaining data.

This problem can be expressed in mathematical form as follows: for a certain data set {*X_i_*}, *I* = 1, 2, …, *N*. The overbound parameters obtained by the MPO and PO methods are ($\sigma_{mpo},\mu_{mpo}$) and ($\sigma_{po},\mu_{po}$), respectively. Under a certain integrity risk (the missed detection probability) $P_{MD}=\beta$, the tolerable maximum deviation is

(27) $$X_{\max}=\underset{\left|X_{b}\right|}{\max}\left\{\left(1-Q\left(\frac{\left|X_{b}\right|-\mu }{\sigma }\right)\right)<\beta \right\},$$

where *Q*(·) is the CDF function of the Gaussian distribution. The larger the *X_max_* value, the greater the maximum allowable deviation, and the stronger the robustness.

We will show that there is a great chance that the tolerable maximum deviation *X_max_mpo_* obtained by the MPO method is greater than the result *X_max_po_* corresponding to the PO method. That is, if a sample with “large deviation” is mistakenly lost in the process of error data collection or model parameter estimation, the parameters obtained by the MPO method can provide a more robust overbound result. 

**Figure 9:**
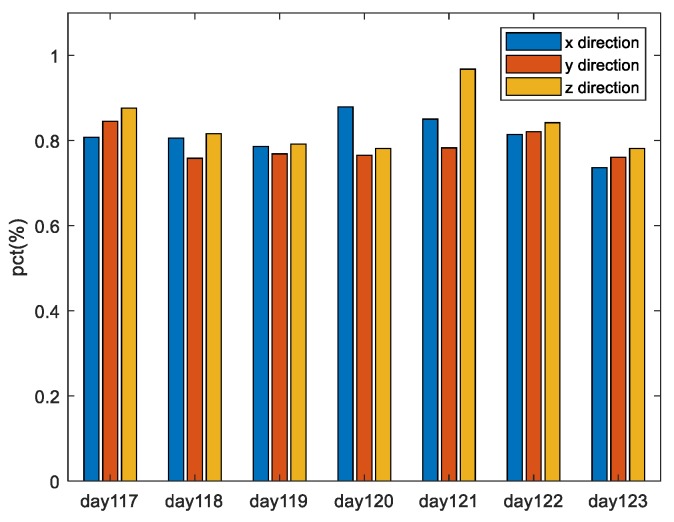
The probability that the MPO tolerable maximum deviation *X_max_mpo_* is greater than the PO *X_max_po_*. It shows that the MPO method has stronger robustness to “large deviations”, with a probability of more than 70%.

Here, we use the experimental settings in Section 4.1 to obtain the ephemeris error results of each satellite in the ECEF coordinate system in the three directions; the time is from 2017day117 to 2017day123. We first use the PO and MPO methods to get the overbound functions and then calculate the “maximum allowable deviation” according to Equation (27). Finally, we calculate the probability that the *X_max_mpo_* is bigger than *X_max_po_* for all the satellites per day. As shown in Figure 9, the horizontal coordinates are the date, and the vertical coordinates represent the probability that the *X_max_mpo_* is greater than the *X_max_po_*. Blue, red, and yellow bars represent the results in the three directions of x, y, and z, respectively. It can be seen that the results are similar in the three directions, and the probability that *X_max_mpo_* is bigger than *X_max_po_* is more than 70% for all days under analysis. That is to say, the MPO method proposed in this paper has “stronger” robustness to large deviations than the PO method, with a probability of more than 70%.

## 5. Conclusions

Based on the analysis of the traditional overbound method and the consideration that the overbound function in the range domain does not need to conform to the characteristics of the probability distribution function, a modified paired overbound method is proposed in this paper. The modified method can realize a more accurate approximation to the actual CDF on the premise of ensuring the integrity of the system, so as to improve the availability of the system. Using the measured ephemeris error data, we compare the proposed MPO method with the traditional PO method in terms of the EVPL index. The experimental results show that by using the modified method, SBAS availability can be improved with a probability of about 99%. In addition, the paper analyzes the robustness of the two methods in the abnormal case of data loss and gives the tolerable maximum deviation under a certain integrity risk. It indicates that the modified paired overbound method has stronger robustness to “large deviations”, with a probability of more than 70%. Besides SBAS, the MPO method proposed in this article can also be used in other conservative error modeling fields related to integrity [39,40,41]. However, it is important to note that the MPO method is only suitable for scenarios that have a linear relationship. For horizontal error modeling, because of the nonlinearity between the observation and the position error, the analysis process in this article is no longer applicable. The next step will be to continue the study on this issue.

## Figures and Tables

**Table 1:** The proportion of users with different rates of growth in Figure 7.

<−0.1	[−0.1,0)	[0,0.1)	[0.1,0.2)	[0.2,0.3)	[0.3,0.4)	[0.4,0.5)	[0.5,0.6)
0.05%	0.99%	4.04%	7.41%	19.17%	29.22%	32.22%	6.90%

**Table 2:** The proportion of users with different rates of growth during 2017day117~2017day123.

	(−0.2,0)	[0,0.2)	[0.2,0.3)	[0.3,0.4)	[0.4,0.5)	[0.5,0.9)
Day117	0	0.048%	0.77%	18.66%	42.41%	38.10%
Day118	0	0.82%	13.53%	55.89%	22.95%	6.80%
Day119	0.29%	13.97%	30.60%	39.24%	14.31%	1.60%
Day120	0	0.58%	4.70%	26.29%	29.51%	38.93%
Day121	1.04%	11.45%	19.17%	29.22%	32.22%	6.90%
Day122	0	0.77%	6.85%	24.67%	30.60%	37.11%
Day123	0.07%	3.20%	9.08%	29.97%	44.23%	13.46%

**Figure A2:**
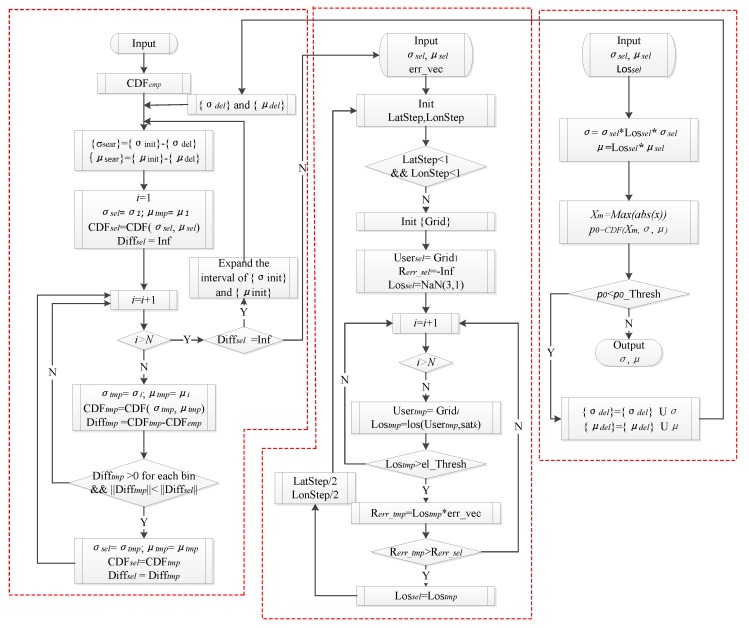
The procedure of parameter estimation. These parts are included: overbounding the error in a single direction, finding the worst user location in the range domain, and hypothesis testing of the results.
